# Coenzyme-A-Responsive Nanogel-Coated Electrochemical Sensor for Osteoarthritis-Detection-Based Genetic Models

**DOI:** 10.3390/gels10070451

**Published:** 2024-07-10

**Authors:** Akhmad Irhas Robby, Songling Jiang, Eun-Jung Jin, Sung Young Park

**Affiliations:** 1 Chemical Industry Institute, Korea National University of Transportation, Chungju 27469, Chungcheongbuk-do, Republic of Korea; irhasakhmad@ut.ac.kr; 2 Department of Chemical & Biological Engineering, Korea National University of Transportation, Chungju 27469, Chungcheongbuk-do, Republic of Korea; 3 Integrated Omics Institute, Wonkwang University, Iksan 54538, Jeonbuk, Republic of Korea; jsl91800@wku.ac.kr; 4 Department of Biological Sciences, College of Health Sciences, Wonkwang University, Iksan 54538, Jeonbuk, Republic of Korea

**Keywords:** coenzyme A, osteoarthritis, manganese oxide, nanogel, polymer dot, electrochemical sensing

## Abstract

An electrochemical sensor sensitive to coenzyme A (CoA) was designed using a CoA-responsive polyallylamine–manganese oxide–polymer dot nanogel coated on the electrode surface to detect various genetic models of osteoarthritis (OA). The CoA-responsive nanogel sensor responded to the abundance of CoA in OA, causing the breakage of MnO_2_ in the nanogel, thereby changing the electroconductivity and fluorescence of the sensor. The CoA-responsive nanogel sensor was capable of detecting CoA depending on the treatment time and distinguishing the response towards different OA genetic models that contained different levels of CoA (wild type/WT, NudT7 knockout/N7KO, and Acot12 knockout/A12KO). The WT, N7KO, and A12KO had distinct resistances, which further increased as the incubation time were changed from 12 h (R_12h_ = 2.11, 2.40, and 2.68 MΩ, respectively) to 24 h (R_24h_ = 2.27, 2.59, and 2.92 MΩ, respectively) compared to the sensor without treatment (R_control_ = 1.63 MΩ). To simplify its application, the nanogel sensor was combined with a wireless monitoring device to allow the sensing data to be directly transmitted to a smartphone. Furthermore, OA-indicated anabolic (*Acan*) and catabolic (*Adamts5*) factor transcription levels in chondrocytes provided evidence regarding CoA and nanogel interactions. Thus, this sensor offers potential usage in simple and sensitive OA diagnostics.

## 1. Introduction

The increasing number of patients with osteoarthritis (OA), characterized by chronic joint and cartilage disabilities, has become a major public health concern [1,2,3]. Finding an effective way to recover from this degenerative disease must be addressed by extensive research on OA pathogenesis, including the development of diagnostic tools and therapeutic approaches [4,5,6]. In studies related to OA, understanding the metabolic pathways and determining the prominent factors regulating OA pathogenesis are crucial for designing effective diagnostic and therapeutic treatment for OA [7,8,9]. OA is characterized by dysfunctional metabolic pathways, which lead to several abnormalities that are absent in healthy cartilage such as the impairment of peroxisomal β-oxidation that triggers the production of higher free fatty acid [10,11,12]. This impairment is also related to the accumulation of acetyl-coenzyme A (acetyl-CoA), obtained from the increasing frequency of CoA-catalyzed glycolysis, which leads to cartilage degradation and joint pain [13,14,15]. In senescent chondrocytes lacking NudT7 knockout (N7KO) and Acot12 knockout (A12KO), changes in acetyl-CoA and CoA concentrations are unique characteristics of OA and cartilage degeneration [16]. Key factors in OA metabolism, such as CoA, can be utilized as indicators of OA progression, which would be beneficial for the early diagnosis of OA and determination of appropriate therapeutic actions. However, there have been only a few reports on the design of a diagnostic platform that can conveniently detect CoA as a benchmark for monitoring OA progression, such as liquid chromatography and the colorimetric and fluorometric approach, which suffers from instability, low sensitivity, and a complex detection procedure [17,18,19,20]. The design of such a sensitive and simple CoA sensor is expected to be advantageous for addressing OA-related issues by providing an intensive monitoring of OA progression.

Biomaterials contribute massively to designing a new diagnosis and therapeutic strategy on bone-related diseases, making approaches more efficient and effective [21,22,23]. In biosensing platform designing, the electrochemical-based biosensor is well known as a facile and commonly used platform for sensing biomarkers because of its reliability and excellent sensitivity [24,25,26,27,28]. Electrochemical biosensors are generally synthesized by coating conductive materials onto the substrate of a sensor (metal nanoparticles, graphene oxide, carbon nanotubes, and conductive polymers) via various methods such as electrospinning and dip coating [29,30,31,32,33,34]. However, these materials suffer from water insolubility and low functionality, which are the main drawbacks in the construction of a sensitive and effective biosensor [35,36,37]. To address this issue, a stimuli-responsive polymer system can be promisingly applied on the biomedical device, including onto the biosensor, owing to the fine-tuning properties by stimuli to tailor the response for a specific use [38]. The polymer dot (PD), one of the stimuli-responsive polymer systems, offers some unique properties, such as biocompatibility, good water solubility, excellent fluorescence property, and electroconductivity, that are beneficial for designing a diagnostic platform [39,40,41,42,43,44]. In terms of functionality, facile functionalization with a broad range of functional groups, such as stimuli-responsive nanoparticles (e.g., MnO_2_) or adhesive moieties (e.g., catechol), is the main advantage that allows the PD to be used as a material for constructing electrochemical biosensors [45,46,47,48]. Considering its versatile functionality and physical form, with features such as its redox sensitivity and incorporation into nanogel, a functionalized PD can be chosen as an alternative material for designing CoA-responsive electrochemical sensors, which are expected to sensitively interact with CoA in OA samples via redox interactions and produce distinct signals for CoA detection. 

In this study, an electrochemical-based CoA sensor was constructed to detect the presence of CoA in OA cartilage by coating a polyallylamine–manganese oxide–polymer dot nanogel (PD@PAH-MnO_2_) onto the electrode substrate. The redox reaction between CoA and the nanogel, which reduces MnO_2_ in nanogel into Mn^2+^, triggers a change in the electroconductivity and other properties (such as fluorescence) of the sensor, which could be used as an indicator for CoA monitoring. The fabricated PD@PAH-MnO_2_ nanogel-coated sensor was expected to sensitively detect CoA in OA genetic models obtained from primary chondrocytes of OA mouse cartilage (wild-type [WT], N7KO, and A12KO). For ease of monitoring, this sensor was combined with a Bluetooth-activated wireless sensing system that allowed the electrochemical detection results to be transmitted and displayed on a smartphone.

## 2. Results and Discussion

### 2.1. Design and Mechanism of CoA-Responsive Nanogel-Coated Sensor for OA Monitoring

**Figure 1:**
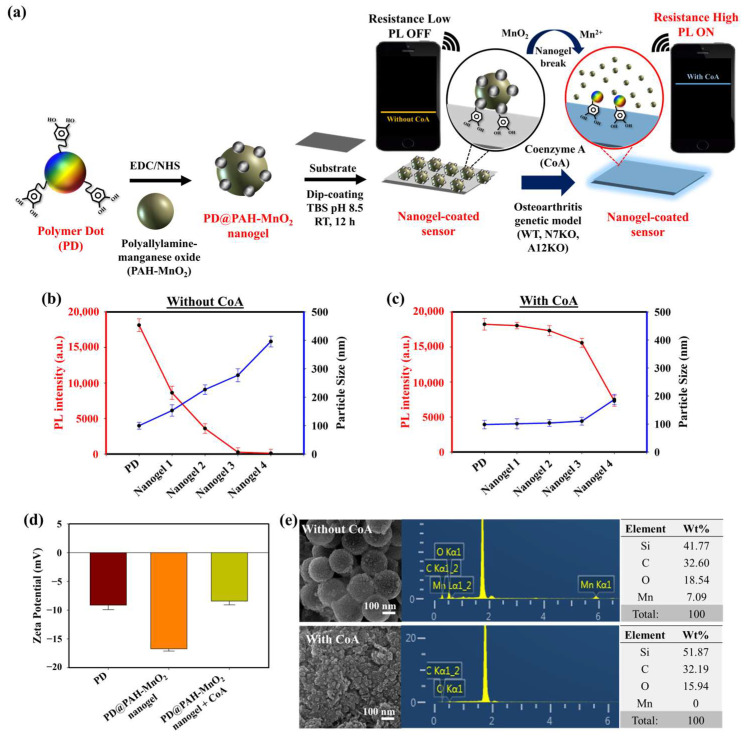
(**a**) Schematic illustration of PD@PAH-MnO_2_ nanogel-coated sensor design and its usage in CoA detection. PL and DLS spectra of different ratios of PD@PAH-MnO_2_ nanogel (**b**) without and (**c**) with CoA treatment (10 mM, 12 h). (**d**) Zeta potential and (**e**) SEM-EDX images of PD@PAH-MnO_2_ nanogel (100:10/nanogel 3) without and with CoA treatment. Notes: nanogel 1 = 100:2.5, nanogel 2 = 100:5, nanogel 3 = 100:10, and nanogel 4 = 100:20 for PD:PAH-MnO_2_.

The presence of CoA and its elevated concentration in OA chondrocytes are prominent biomarkers of OA progression. An electrochemical sensing approach was developed to sensitively monitor CoA in OA chondrocytes using a CoA-responsive nanogel coated on the surface of an electrode. The CoA-responsive nanogel (PD@PAH-MnO_2_ nanogel) was synthesized by conjugating PAH-MnO_2_ and catechol-functionalized PD via an EDC-NHS coupling reaction followed by surface coating on the electrode (Si wafer), where the PAH-MnO_2_ interacted with CoA via redox interaction, and the PD provided distinct fluorescence properties and promoted good nanogel coatability on the electrode surface due to the presence of catechol moieties. The sensing mechanism of this nanogel sensor relied on the change in conductivity owing to the redox interaction between MnO_2_ in the nanogel and CoA in OA chondrocytes, thereby converting MnO_2_ into Mn^2+^, which was used as an indicator for CoA detection. In OA progression, the concentrations of other natural thiol species similar to CoA, such as glutathione (GSH), are dramatically depleted and found to be in trace amounts in OA joints, which is contrast to the abundant elevated CoA concentration. Owing to this trace concentration, the influence of GSH on CoA detection can be neglected [49,50,51]. In this study, the breakage of MnO_2_ by CoA also altered the fluorescence properties of the nanogel. The fluorescence of the nanogel was initially quenched owing to the presence of MnO_2_ that triggered fluorescence resonance energy transfer (FRET) between the PD and MnO_2_, the degree of fluorescence quenching of which was higher as the amount of MnO_2_ was increased. When CoA was present, the fluorescence of nanogel was recovered as the FRET effect was reduced after MnO_2_ breakage [45]. This fluorescence recovery could be more intense as the CoA concentration was increased, such as in the higher CoA levels of N7KO and A12KO compared to the WT primary cell. Based on these phenomena, the PD@PAH-MnO_2_ nanogel-coated sensor could respond to different levels of expressed CoA in OA genetic models such as the WT, N7KO, and A12KO models. For ease of monitoring, an Arduino-based wireless device arrangement was connected to the sensor to read and transmit the electronic signal of CoA detection and was further displayed on a smartphone via Bluetooth activation (Figure 1a).

### 2.2. Effect of CoA on PD@PAH-MnO_2_ Nanogel Properties

**Figure 2:**
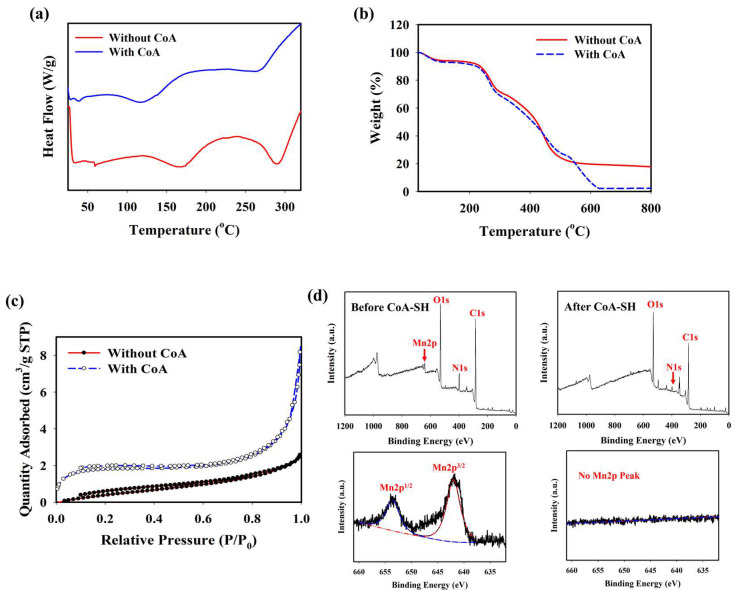
(**a**) DSC, (**b**) TGA, (**c**) BET, and (**d**) XPS measurements (survey and narrow scan) of PD@PAH-MnO_2_ nanogel without and with CoA treatment (10 mM, 12 h).

The optimum condition for designing a CoA-responsive nanogel was decided by varying the ratio of the PD and PAH-MnO_2_. Nanogels with different ratios (PD: PAH-MnO_2_ = 100:2.5/nanogel 1, 100:5/nanogel 2, 100:10/nanogel 3, and 100:20/nanogel 4) were treated with a CoA solution (10 mM) and the changes in the physical properties of the nanogels affected by CoA were evaluated. Before CoA treatment, the average size of the nanogel was higher than that of the PD only (98.6 nm), and it increased as the ratio of PAH-MnO_2_ increased from nanogel 1 to nanogel 4 (156.8, 213.7, 283.9, and 392.4 nm, respectively). A higher amount of MnO_2_ also caused significant fluorescence quenching in the nanogel as FRET occurred between the PD and PAH-MnO_2_ (Figure 1b). When CoA was introduced into nanogel 1–4, a decrease in nanogel size was observed for all nanogel ratios (101.6, 110.3, 118.0, and 188.5 nm, respectively) whereas the fluorescence intensity was significantly recovered for all nanogels (Figure 1c). The change in the size and fluorescence properties of the nanogel after CoA treatment clearly indicated the decomposition efficacy of MnO_2_ nanosheets into Mn^2+^ by CoA, leading to reduced nanogel sizes and a lower FRET effect. Based on these data, the ratio of nanogel 3 was chosen as the optimum condition and used for further experiments. A change in the zeta potential was also observed after treatment with CoA. The zeta potentials of the PD and PD@PAH-MnO_2_ nanogel were observed at −9.14 and −16.73 mV, respectively. After reaction with 10 mM CoA, the zeta potential of the PD@PAH-MnO_2_ nanogel changed to −7.75 mV owing to the effect of MnO_2_ decomposition into Mn^2+^ (Figure 1e). SEM-EDX analysis further revealed an alteration in the nanogel morphology between pre- and post-treatment with CoA. The PD@PAH-MnO_2_ nanogel was round in shape, with a high amount of Mn present in the nanogel before CoA treatment. However, this structure was broken into smaller particles as CoA was exposed to the nanogel, and the amount of Mn decreased dramatically, indicating the decomposition of the nanogel in the presence of CoA, particularly because of the cleavage of MnO_2_ in the nanogel (Figure 1e). This structural change in the nanogel was further confirmed by assessing its thermal properties using the DSC and TGA profiles. As shown in the DSC graph (Figure 2a), *T_m_* peaks of the nanogel shifted before (169.7°C and 291.5 °C) and after CoA treatment (117.3 °C and 264.1 °C) (Figure 2a). The TGA profiles also showed the distinct thermal features of the nanogel pre- and post-treatment with CoA, with the post-treated hydrogel exhibiting additional degradation between 535.1 °C and 623.7 °C (Figure 2b). Moreover, the change in the pore density of the nanogel indicated the effect of CoA. The pore density of the nanogel without CoA was 2.6291 m^2^/g whereas the pore density with CoA treatment was 6.9718 m^2^/g (Figure 2c). An increase in the surface area correlated with a decrease in the nanogel size after treatment owing to the decomposition of MnO_2_ by CoA. In addition, XPS analysis revealed the elemental composition and the disappearance of MnO_2_ after CoA treatment. The survey scan XPS of the PD@PAH-MnO_2_ nanogel showed the presence of peaks centered at 283.7, 398.7, 531.5, and 642.1 eV corresponding to C1s, N1s, O1s, and Mn2p, respectively. Furthermore, the Mn2p peak disappeared after CoA treatment. The narrow scan clearly showed the disappearance of Mn2p^3/2^ and Mn2p^1/2^ at 642.1 and 653.5 eV on the electrode surface as a result of the reaction with CoA (Figure 2d) [45]. Because of the presence of catechol moieties as an adhesive functional group in the nanogel, the PD@PAH-MnO_2_ nanogel was easily coated on the Si wafer surface to construct CoA-responsive nanogel sensor. The successful coating and coating stability of the PD@PAH-MnO_2_ nanogel was assessed via contact angle and conductivity measurements of PD@PAH-MnO_2_ nanogel-coated Si wafer (Figure S1). The contact angle of Si wafer (30.2°) was changed after coating with the PD@PAH-MnO_2_ nanogel (43.2°). This nanogel coating was stable even after being soaked in the cell media for 24 h (43.1°), confirming the coating stability of nanogel sensor. The same change was also observed from the resistance of Si wafer before (13.10 MΩ) and after nanogel coating (1.55 MΩ), with a negligible effect of cell media on the nanogel-coated Si wafer resistance (1.56 MΩ), confirming the excellent adhesion and coating stability of the nanogel on the fabricated sensor.

### 2.3. Electrochemical Detection of CoA in OA Genetic Models Using PD@PAH-MnO_2_ Nanogel-Coated Sensor

**Figure 3:**
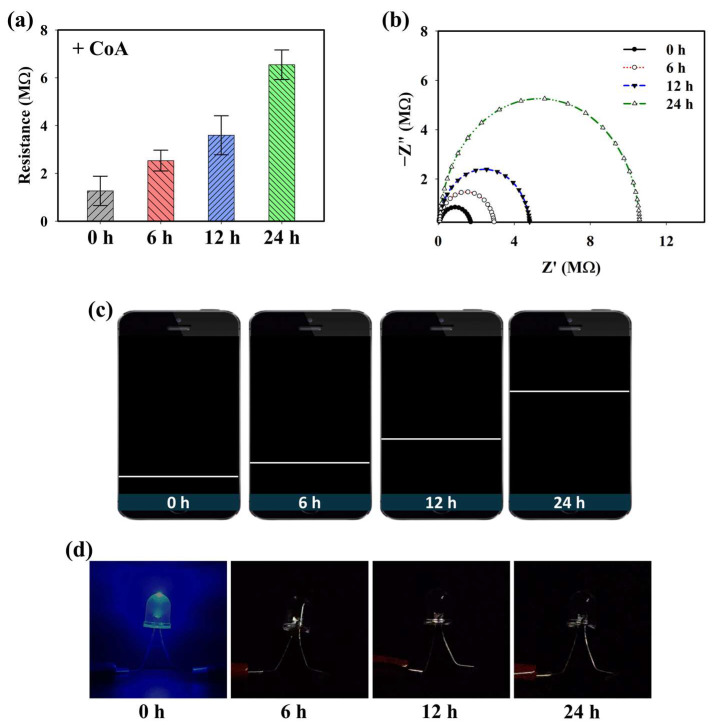
(**a**) Sourcemeter data, (**b**) EIS profiles, (**c**) wireless sensing display, and (**d**) simple LED experiment of PD@PAH-MnO_2_ nanogel-coated sensor treated with 10 mM CoA for different duration (0, 6, 12, 24 h).

Because the PD@PAH-MnO_2_ nanogel contains catechol moieties that provide adhesive properties, it can be coated on the electrode surface (Si wafer) to construct a CoA-responsive sensor. After coating, the ability of the PD@PAH-MnO_2_ nanogel-coated sensor to respond to CoA was evaluated by observing the change in the electroconductivity of the sensor depending on the duration of CoA treatment. Sourcemeter measurements clearly showed a change in the resistance, particularly as the treatment time increased (Figure 3a). The resistance was increased from 1.27 MΩ at 0 h to 2.53, 3.60, and 6.55 MΩ at 6, 12, and 24 h of CoA treatment. This result indicates that the fabricated nanogel-coated sensor was capable of sensitively detecting CoA, with a notable change in resistance measured as the incubation time varied owing to the increased cleavage of MnO_2_. These data were further confirmed by EIS measurements (Figure 3b). The Nyquist plot shows the gradual change in impedance that occurred in the PD@PAH-MnO_2_ nanogel-coated sensor when interacting with CoA. As the interaction time increased, the impedance also increased, indicating that the change in electroconductivity was affected by the breakage of the nanogel. To develop a simple approach for CoA detection, a wireless sensing system was utilized by combining a Bluetooth-assisted microcontroller with a PD@PAH-MnO_2_ nanogel-coated sensor. Using this system, sensing data were transmitted and displayed on a smartphone as a resistance graph by activating a Bluetooth connection. As shown in Figure 3c, the resistance graph displayed on a smartphone closely correlated with the sourcemeter and EIS data wherein the resistance increased as the treatment time increased, confirming the electrochemical detection capability of the PD@PAH-MnO_2_ nanogel-coated sensor. Changes in the electrochemical properties before and after CoA interaction were also observed by CV measurements (Figure S2). The nanogel sensor without CoA treatment showed notable oxidation peaks at 0.62 V and 1.32 V. However, with CoA treatment, the current density of the nanogel sensor was lower than that without CoA, with the disappearance of a peak at 0.62 V. This change confirmed that the breakage of MnO_2_ in the nanogel was triggered by CoA, which affected the electrochemical properties of the PD@PAH-MnO_2_ nanogel-coated sensor. In addition, a simple LED experiment demonstrated the enhanced resistance of the nanogel sensor treated with CoA as the light intensity dramatically decreased compared to that of the control (0 h) (Figure 3d).

**Figure 4:**
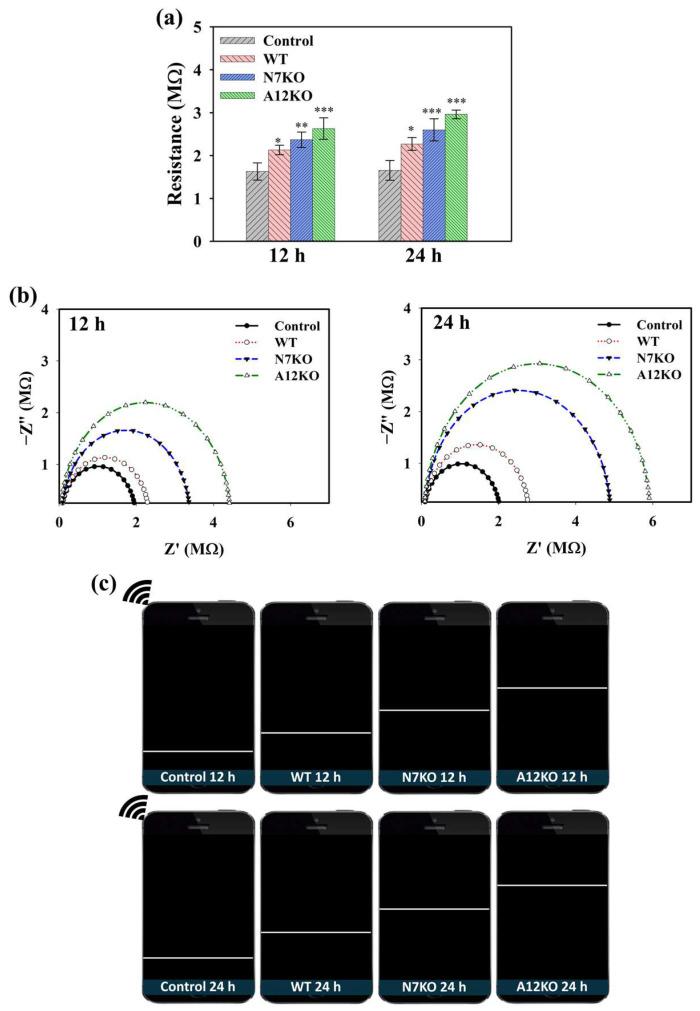
(**a**) Sourcemeter data (* = *p* < 0.1, ** = *p* < 0.01, *** = *p* < 0.001), (**b**) EIS profiles, and (**c**) wireless sensing display of PD@PAH-MnO_2_ nanogel-coated sensor treated with OA genetic models from primary chondrocytes (WT, N7KO, and A12KO) for 12 and 24 h (*n* = 6).

CoA plays a crucial role in the generation of acetyl-CoA in cells during OA pathogenesis. CoA catalyzes acetyl-CoA production during glycolysis, and accumulated acetyl-CoA can be used as an indicator of OA. Hence, the performance of the PD@PAH-MnO_2_ nanogel-coated sensor in detecting CoA was further evaluated using an in vitro culture of primary articular chondrocyte-isolated OA genetic models (WT, N7KO, and A12KO) that express high levels of CoA. After PD@PAH-MnO_2_ nanogel-coated sensor was seeded by each model for different incubation time, the change in electroconductivity was measured and compared with the nanogel sensor without any samples (control). Based on sourcemeter measurement, the resistance of the PD@PAH-MnO_2_ nanogel-coated sensor increased after treatment with the WT, N7KO, and A12KO, which further increased as the treatment time increased from 12 h (2.11, 2.40, and 2.68 MΩ, respectively) to 24 h (2.27, 2.59, and 2.92 MΩ, respectively) compared to the control (12 h = 1.63 MΩ, 24 h = 1.65 MΩ) (Figure 4a). Interestingly, the resistance values of N7KO and A12KO were higher than those of the WT owing to the higher level of CoA in N7KO and A12KO, allowing more breakage of MnO_2_, thereby increasing the resistance of the sensor. Based on the statistical analysis of *p* values, the differences between the control and OA genetic-model-treated nanogel sensor were significant, particularly during the detection of N7KO and A12KO after 24 h (*p* < 0.001, *n* = 6), confirming the detection capability of the sensor towards OA. The change in resistance was also analyzed using EIS (Figure 4b). An increasing trend of the Nyquist plot for the WT-, N7KO-, and A12KO-treated nanogel sensors clearly indicated the difference in impedance affected by CoA, in which enhanced impedance was observed at 24 h of incubation compared to that at 12 h of incubation. Moreover, the patterns of enhanced resistance displayed on the smartphone via the wireless sensing system closely correlated with the data obtained using the sourcemeter and EIS, revealing the performance of the PD@PAH-MnO_2_ nanogel-coated sensor in detecting different CoA levels in various genetic models of OA (Figure 4c).

### 2.4. Cellular Analysis and Transcriptional Studies of Anabolic–Catabolic Factors in OA Genetic Models

**Figure 5:**
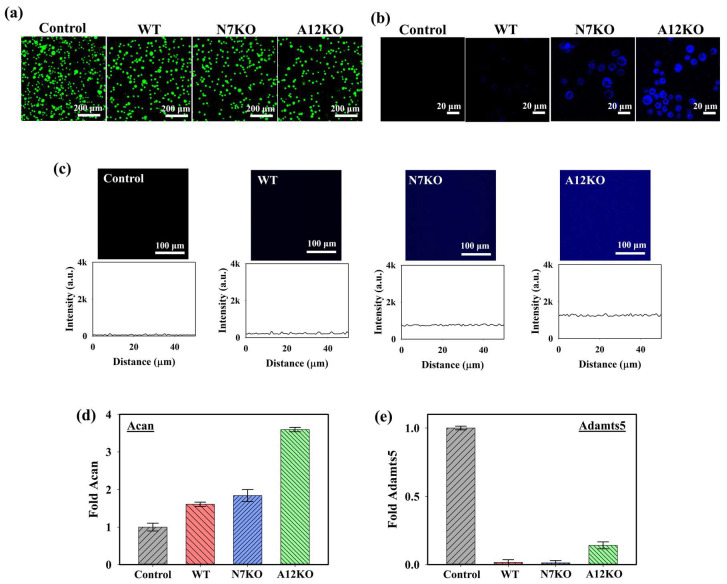
(**a**) Live and dead staining assay (green = live, red = dead), (**b**) cellular uptake, and (**c**) fluorescence recovery of the PD@PAH-MnO_2_ nanogel-coated sensor treated with WT, N7KO, and A12KO (24 h). Transcriptional level of (**d**) anabolic factor aggrecan (*Acan*) and (**e**) catabolic factor *Adamts5* genes in WT, N7KO, and A12KO after seeding on PD@PAH-MnO_2_ nanogel-coated sensor.

To assess the cytotoxicity of the PD@PAH-MnO_2_ nanogel-coated sensor on the cells, live and dead staining analyses were performed under a confocal microscope using annexin-V (live, green fluorescence) and propidium iodide (dead, red fluorescence). As shown in the confocal images in Figure 5a, the PD@PAH-MnO_2_ nanogel-coated sensor exhibited low cytotoxicity towards cells of the OA genetic models after seeding with the WT, N7KO, and A12KO for 24 h as indicated by the dominant green fluorescence in the obtained cell images. This result was consistent with various reports that revealed the excellent biocompatibility of the PD and MnO_2_, which makes those nanomaterials suitable to be used for biomedical applications [52,53,54,55]. Furthermore, the cellular uptake of PD@PAH-MnO_2_ in the WT, N7KO, and A12KO was investigated (Figure 5b). Distinct fluorescence intensities were observed in the WT, N7KO, and A12KO, with A12KO exhibiting the brightest fluorescence, indicating the differential fluorescence recovery of nanogel uptake by cells, as the levels of CoA were different for the WT, N7KO, and A12KO. The interaction between the WT, N7KO, and A12KO PD@PAH-MnO_2_ nanogel-coated sensors was also evaluated by observing the fluorescence recovery on the substrate (for this experiment, PET film) coated with the nanogel, which reflected MnO_2_ breakage in the nanogel. As shown in Figure 5c, distinct fluorescence recovery was observed in the PD@PAH-MnO_2_ nanogel-coated PET treated with each OA genetic model, particularly for substrates incubated with A12KO, which showed the most significant fluorescence recovery compared to the WT and N7KO. These findings confirmed the reduced FRET effect in the nanogel as MnO_2_ was cleaved by CoA in the OA genetic models. Moreover, to understand the interaction between the PD@PAH-MnO_2_ nanogel and CoA in cells during detection, the PD@PAH-MnO_2_ nanogel was expected to scavenge CoA, and the transcriptional levels of anabolic–catabolic factors in the OA genetic models were determined. In OA cartilage chondrocytes, the levels of anabolic factors such as aggrecan (*Acan*) are highly suppressed. In contrast, catabolic factors such as *Adamts-5*, which play a vital role in cartilage matrix degradation, are expressed in OA. After incubation with the PD@PAH-MnO_2_ nanogel-coated sensor, *Acan* expression in the WT, N7KO, and A12KO was significantly enhanced, particularly in the A12KO (Figure 5d). In contrast, the levels of *Adamts5* expression in the WT, N7KO, and A12KO were dramatically suppressed (Figure 5e). The increased and decreased levels of *Acan* and *Adamts5* revealed lower concentrations of CoA owing to exposure to the PD@PAH-MnO_2_ nanogel-coated sensor, which reduced acetyl-CoA accumulation in the cells and further altered the levels of *Acan* and *Adamts5*. These results demonstrate the capability of the PD@PAH-MnO_2_ nanogel-coated sensor for sensitive electrochemical-based OA detection.

## 3. Conclusions

A CoA-responsive PD@PAH-MnO_2_ nanogel sensor was successfully fabricated to sensitively detect CoA levels in OA genetic models via the electrochemical approach. The PD@PAH-MnO_2_ nanogel sensor responded to CoA via a redox reaction that converted MnO_2_ into Mn^2+^ triggered by CoA, thereby changing the electroconductivity, fluorescence, and other properties of the nanogel sensor. The PD@PAH-MnO_2_ nanogel sensor exhibited sensitivity towards CoA as indicated by the change in resistance before and after treatment with CoA depending on the reaction time. When the sensor was used for detection in the OA genetic models, significant differences were observed in resistance values between WT, N7KO, and A12KO. In addition, the continuous treatment times from 12 h (R_12h_ = 2.11, 2.40, and 2.68 MΩ, respectively) to 24 h (R_24h_ = 2.27, 2.59, and 2.92 MΩ, respectively) altered the resistance of the sensor. Furthermore, the resistance obtained during CoA detection can be transmitted and displayed on a smartphone via a Bluetooth connection in a wireless sensing device, which simplifies the OA sensing application. In addition, the changes in anabolic–catabolic factors (*Acan* and *Adamts5*) in WT, N7KO, and A12KO after exposure to the PD@PAH-MnO_2_ nanogel sensor demonstrated the interaction between CoA and the PD@PAH-MnO_2_ nanogel. This evidence suggests that the PD@PAH-MnO_2_ nanogel sensor has potential applications for the sensitive and simple detection of OA.

## 4. Materials and Methods

### 4.1. Materials

Alginic acid sodium salt, coenzyme A (CoA), dopamine hydrochloride, (*N*-morpholino)ethanesulfonic acid (MES), potassium permanganate (KMnO_4_), poly(allylamine hydrochloride) (PAH), trizma base, ethylcarbodiimide hydrochloride (EDC), and *N*-hydroxysuccinimide (NHS) were purchased from Sigma-Aldrich (St. Louis, MO, USA). Phosphate buffered saline (PBS) was purchased from Bioneer Corp. (Daejeon, Republic of Korea). P-type Silicon wafers were obtained from Silicon Technology Corporation (Daejon, Republic of Korea). Trypsin-ethylenediaminetetraacetic acid (trypsin-EDTA, 0.03% *w*/*v*), fetal bovine serum (FBS), penicillin–streptomycin, and cell medium were obtained from Gibco BRL (New York, NY, USA). Live and dead cell staining systems (live: annexin V-FITC, dead: propidium iodide) were purchased from Life Technologies (Carlsbad, CA, USA).

### 4.2. Characterizations

The diameter and zeta potential of the nanogel were analyzed using a Zetasizer Nano (Malvern Panalytical, Kassel, Germany). The photoluminescence (PL) characteristics of the nanogel were assessed using a fluorescence spectrometer (L550B; Perkin Elmer, Shelton, CT, USA). Scanning electron microscopy–electron dispersive X-ray spectroscopy (SEM-EDX) was performed using a JSM-6700F instrument (JEOL, Tokyo, Japan). Thermal gravimetric analysis (TGA) and differential scanning calorimetry (DSC) were performed using a TGA 8000 and DSC4000 instrument, respectively (Perkin Elmer, Shelton, CT, USA). Confocal imaging was performed using a confocal microscope (ECLIPSE Ti2-E, Nikon, Tokyo, Japan). The surface area of the nanogel was measured using a Tristar II 3020 (Micromeritics, Norcross, GA, USA). Structural characterization of the nanogel was performed using X-ray photoelectron spectroscopy (XPS; Omicron ESCALAB, Berlin, Germany). Electrochemical impedance spectrometry (EIS) and cyclic voltammetry (CV) measurements were performed using an electrochemical workstation (CS350, CorrTest Instruments, Wuhan, China). A 2-electrode DC system sourcemeter (Keithley 2450, Tektronik, Cleveland. OH, USA) was used to measure the resistance of the nanogel sensors. An Arduino Uno microcontroller (ATmega328P Processor, sensing part), a Bluetooth module (AppGosu, Somerville, MA, USA), and a smartphone were used as the wireless sensing system for real-time data monitoring.

### 4.3. Synthesis of CoA-Responsive Nanogel (PD@PAH-MnO_2_)

CoA-responsive nanogel was synthesized by reacting PD with PAH-MnO_2_. In brief, PD containing catechol moieties was obtained from the hydrothermal carbonization of alginate-dopamine as described in a previous report [39]. PAH-MnO_2_ was then synthesized by reacting 57.4 mg KMnO_4_ with 60 mg PAH in 70 mL double-distilled water (DDW), followed by the addition of 120 mL MES buffer solution (0.1 M, pH 6.0), and allowed to react for 2 h under sonication. The mixture was dialyzed against DDW (cutoff: 3500 Da) and freeze-dried. The obtained PAH-MnO_2_ was then reacted with PD at different ratios (PD:PAH-MnO_2_ = 100:2.5/nanogel 1, 100:5/nanogel 2, 100:10/nanogel 3, and 100:20/nanogel 4) via the EDC-NHS coupling reaction at room temperature for 24 h [40]. Subsequently, the mixtures were dialyzed against DDW (cut-off: 3500 Da) and freeze-dried. To assess the PD@PAH-MnO_2_ nanogel structure in the absence and presence of CoA, the nanogel structural integrity and properties was analyzed by exposing the PD@PAH-MnO_2_ nanogel with different PD:PAH-MnO_2_ ratios (nanogel 1–4) to a CoA solution (10 mM) for 12 h at room temperature. The changes in the nanogel size and surface area were measured using dynamic light scattering (DLS) and Brunauer–Emmett–Teller (BET) methods whereas the structural and morphological changes in the CoA-treated nanogel were observed using SEM-EDX and XPS. The changes in thermal properties of nanogel were also observed using DSC (25–300 °C, 10 °C/min, N_2_ atmosphere) and TGA (25–800 °C, 10 °C/min, N_2_ atmosphere).

### 4.4. Synthesis of CoA-Responsive PD@PAH-MnO_2_ Nanogel-Coated Sensor

A CoA-responsive nanogel sensor was fabricated by coating the PD@PAH-MnO_2_ nanogel onto an electrode substrate (Si wafer, 1 cm × 1 cm) via the dip-coating method. Briefly, an Si wafer was soaked in 2 mg/mL PD@PAH-MnO_2_ nanogel (pH 8.5) and allowed to react overnight at room temperature. The coated surfaces were washed with DDW and dried before use. 

### 4.5. Electrochemical-Based Detection of CoA Using PD@PAH-MnO_2_ Nanogel-Coated Sensor

The electrochemical sensing capability of the PD@PAH-MnO_2_ nanogel-coated sensor towards CoA was evaluated by measuring the resistance using a 2-electrode DC system sourcemeter, wireless sensing device, and 3-electrode system EIS, as well as by observing the I–V profiles using CV. The PD@PAH-MnO_2_ nanogel-coated sensor was treated with CoA solution (10 mM) for different durations (0, 6, 12, and 24 h). The nanogel-coated sensor was washed with DDW and dried using an air compressor before the electrochemical measurements. The resistance was measured by connecting the nanogel-coated sensor to a sourcemeter (2-electrode DC setting). For wireless sensing, the nanogel-coated sensor was combined with a wireless sensing system comprising a Bluetooth module and a microcontroller circuit using alligator clips. By turning on the Bluetooth on a smartphone, the sensing data (shown as a resistance graph) can be transmitted and displayed on a smartphone. Furthermore, EIS analysis was conducted using the nanogel-coated sensor as the working electrode, Ag/AgCl as the reference electrode, a Pt wire as the counter electrode, and PBS (pH 7.4) as an electrolyte. The frequency ranged from 10^4^–10^−1^ Hz with −1.2 V of DC bias at 25 °C. The CV measurement was also conducted using similar set-up as the EIS measurement (working electrode: PD@PAH-MnO_2_ nanogel-coated sensor, reference electrode: Ag/AgCl, counter electrode: Pt wire, electrolyte: PBS pH 7.4), with voltage in the range −1.5–1.5 V at a scan speed of 50 mV/s.

### 4.6. Sensing Ability of PD@PAH-MnO_2_ Nanogel-Coated Sensor towards In Vitro OA Genetic Models

The in vitro OA genetic models (WT, N7KO, and A12KO) were obtained by isolating immature articular chondrocyte (iMAC) primary cultures from postnatal day 5–6 articular cartilage of mice by tibial plateau and femoral condyle dissection. Digestion of cartilage was performed using collagenase D solution (3 mg/mL, Roche, Basel, Switzerland, 11088858001) for 45 min, followed by transfer to a culture dish containing collagenase D solution (0.5 mg/mL) and incubation at 37 °C for 12 h. After filtration (70 μm cell strainer), primary iMACs were cultured in Dulbecco’s modified Eagle’s medium (low glucose, 1 g/L) containing penicillin–streptomycin and FBS (10%) at 37 °C for 5 days in the presence of 5% CO_2_. For electrochemical sensing experiments, cultured WT, N7KO, and A12KO were seeded onto the PD@PAH-MnO_2_ nanogel-coated sensor for 12 h and 24 h at 37 °C, then washed and dried before measurement. To evaluate the significance of the sensing data compared to the control, the obtained sensing results were further statistically analyzed using Student’s *t*-test to determine *p* values (* = *p* < 0.1, ** = *p* < 0.01, *** = *p* < 0.001), and number of replications of each sample was 6 (*n* = 6).

### 4.7. Confocal Imaging of PD@PAH-MnO_2_ Nanogel-Coated Sensor

PD@PAH-MnO_2_ nanogel was coated on the polyethylene terephthalate (PET) surface for fluorescence observation after detecting CoA using dip-coating method mentioned in Section 4.5. The PD@PAH-MnO_2_ nanogel-coated PET surface was then seeded with WT, N7KO, and A12KO for 12 h at 37 °C. Prior to fluorescence imaging, the coated surfaces were washed with DDW and dried using an air compressor. The fluorescence of PD@PAH-MnO_2_ nanogel-coated PET surface was observed under confocal microscope.

## Data Availability

The data presented in this study are available upon request from the corresponding author for privacy reasons.

## Supplementary Materials

The following supporting information can be downloaded from https://www.mdpi.com/article/10.3390/gels10070451/s1, Figure S1: Cyclic voltammogram of the PD@PAH-MnO_2_ nanogel-coated sensor without and with CoA treatment. Figure S2: Cyclic voltammogram of PD@PAH-MnO_2_ nanogel-coated sensor without and with CoA treatment (10 mM, 12 h).
