# Supplementary material for: Coenzyme-A-Responsive Nanogel-Coated Electrochemical Sensor for Osteoarthritis-Detection-Based Genetic Models
Source: Gels. 2024 Jul 10;10(7):451. doi: 10.3390/gels10070451 (PMC11276253; doi:10.3390/gels10070451)
Supplement: Supplementary file 1 [file gels-10-00451-s001.zip › gels-3089543-supplementary.pdf]

Supporting Information

Coenzyme-A-Responsive Nanogel-Coated Electrochemical Sensor for Osteoarthritis-Detection-Based Genetic Models

Akhmad Irhas Robby [1,2,†], Songling Jiang [3,†], Eun-Jung Jin [3,4,*] and Sung Young Park [1,2,*]

1 Chemical Industry Institute, Korea National University of Transportation, Chungju 27469, Chungcheongbuk-do, Republic of Korea; irhasakhmad@ut.ac.kr
2 Department of Chemical & Biological Engineering, Korea National University of Transportation, Chungju 27469, Chungcheongbuk-do, Republic of Korea
3 Integrated Omics Institute, Wonkwang University, Iksan 54538, Jeonbuk, Republic of Korea; jsl91800@wku.ac.kr
4 Department of Biological Sciences, College of Health Sciences, Wonkwang University, Iksan 54538, Jeonbuk, Republic of Korea
* Correspondence: jineunjung@wku.ac.kr (E.-J.J.); parkchem@ut.ac.kr (S.Y.P.)
† These authors contributed equally to this work.

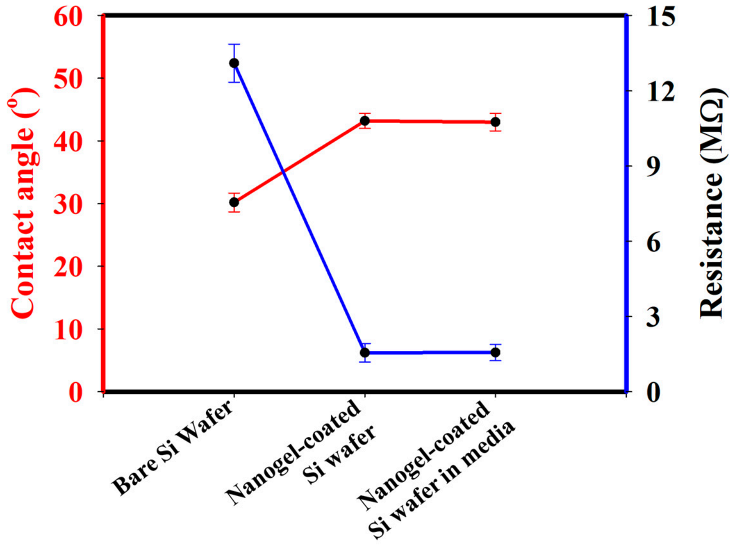

Figure S1. Coating stability of PD@PAH-MnO_2 nanogel-coated sensor.

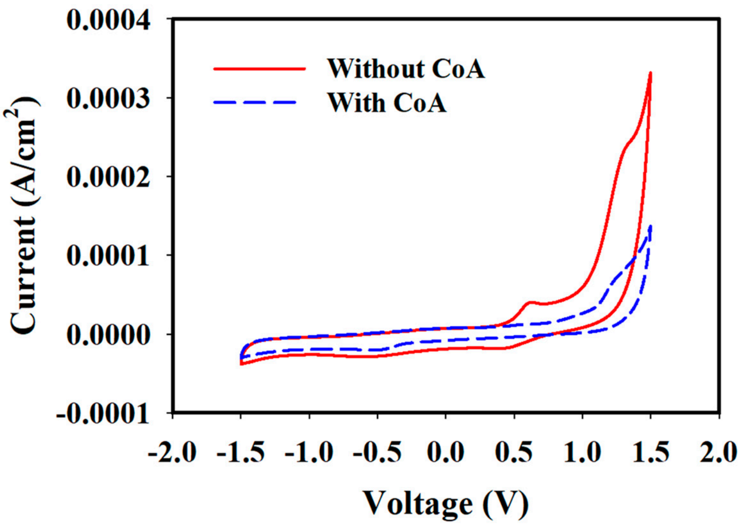

Figure S2. Cyclic voltammogram of PD@PAH-MnO_2 nanogel-coated sensor without and with CoA treatment (10 mM, 12 h).
